# Evidence of synergy with combined BRAF-targeted therapy and immune checkpoint blockade for metastatic melanoma

**DOI:** 10.4161/21624011.2014.954956

**Published:** 2014-12-08

**Authors:** Zachary A Cooper, Alexandre Reuben, Rodabe N Amaria, Jennifer A Wargo

**Affiliations:** 1 Division of Surgical Oncology; The University of Texas MD Anderson Cancer Center; Houston, TX USA; 2 Genomic Medicine; The University of Texas MD Anderson Cancer Center; Houston, TX USA; 3 Department of Melanoma Medical Oncology; The University of Texas MD Anderson Cancer Center; Houston, TX USA

**Keywords:** BRAF, immune checkpoint, PD-1, PD-L1, melanoma

## Abstract

Significant advances in the treatment of melanoma have been made with BRAF-targeted therapy and immune checkpoint blockade, and these strategies are now being combined empirically in clinical trials. Potential synergy is demonstrated in murine models and in analysis of longitudinal biopsies from patients on trial, however important questions remain regarding toxicity, optimal timing and sequence of therapy.

In recent years, 2 significant advances in the treatment of metastatic melanoma have emerged: the use of BRAF targeted therapy and immune checkpoint blockade. Treatment with targeted therapy results in rapid responses in the majority of patients,^1,2^ although resistance to therapy remains predictable and almost universal.^1,2^ Conversely, treatment with immune checkpoint inhibitors results in lower overall response rates, though responses tend to be more durable.^3,4^

There is growing evidence that oncogenic BRAF contributes to immune escape, and that targeting this mutation with BRAF inhibitors (BRAFi) may make melanoma tumors more immunogenic.^5-8^ This has been seen *in vitro* where treatment with a BRAFi is associated with an up-regulation of melanoma differentiation antigens melanoma differentiation antigens and enhanced recognition by antigen-specific T cells.^6^ Importantly this enhanced immunogenicity has also been observed *in vivo*, where treatment of patients with BRAFi monotherapy or combined BRAF/MEK inhibition is associated with a more favorable tumor microenvironment within 10-14 d of initiation of therapy with enhanced melanoma antigen expression, increased CD8^+^ T-cell infiltrate, increased T-cell activation markers, and a decrease in the levels of immunosuppressive cytokines and the angiogenic factor VEGF.^5,7,8^ However, there is a concurrent increase in the expression of immunomodulatory molecules including Programmed Cell Death 1 (PD-1) on the infiltrating T cells (probably relating to their activation status), and Programmed Death 1 Ligand (PD-L1) in the tumor microenvironment.^8^ Together, these data suggest that there is an immune response to BRAFi, though this may be attenuated early in the course of therapy due to changes in the tumor microenvironment.

The concept of combining BRAF-targeted therapy and immunotherapy is being empirically tested in currently enrolling clinical trials. Response data is not mature, and there have been significant adverse events including hepatotoxicity and colitis with combined regimens in these early trials.^9,10^ To optimally study these combinations, we must incorporate longitudinal tissue analyses in human clinical trials to better understand changes within the tumor microenvironment. We must also try to better understand the mechanism of responses through pre-clinical models. We recently published *in vivo* and *in vitro* findings demonstrating evidence of potential synergy with combined BRAF-targeted therapy and immune checkpoint blockade.^11^

We began by analyzing longitudinal biopsies from a patient who received combined BRAF-targeted therapy and ipilimumab. Tumor biopsies demonstrated an early and transient CD8^+^ T-cell response that was restored after the addition of immune checkpoint blockade and persisted for several months.^11^ Next, we studied this in a BRAF-mutant murine model of melanoma. We utilized a transplantable murine melanoma model in C57BL/6 mice developed from an inducible Tyr:CreER; Braf^CA^; Pten^lox/lox^ murine model^12^ and demonstrated a dose dependent response to BRAFi and an increase in CD8^+^ T-cell density and cytokine production.^11^ Additionally, CD8^+^ depletion demonstrated a critical role for CD8^+^ T cells in response to BRAFi. In these studies, we chose to combine BRAF-targeted therapy with immune checkpoint blockade against the PD-1 axis, as a more favorable toxicity profile is seen with these drugs as compared with CTLA-4 blockade. Treatment with BRAFi monotherapy resulted in a modest increase in T-cell infiltrate and a significant (but small) improvement in survival over control mice. Conversely, treatment with monotherapy using blocking antibodies against PD-1 or PD-L1 resulted in a modest increase in T-cell infiltrate and no difference in survival. However, mice that were treated with combined BRAFi and either PD-1 or PD-L1 blocking antibody demonstrated a dramatic increase in infiltrating T cells as well as enhanced survival associated with abrogated melanoma growth.^11^ Mechanistic studies demonstrated that infiltrating T cells isolated from tumors of mice treated with combined BRAFi and PD-1 or PD-L1 blockade produced more interferon γ (IFNγ) and tumor necrosis factor α (TNFα) than those T cells arising from tumors of mice treated with BRAFi alone.^11^ These findings suggest that CD8^+^ T cells are recruited in the setting of BRAF-targeted therapy, but that they are maintained in a suppressed state by the tumor microenvironment. However these T cells can be activated via the addition of immune checkpoint blockade leading to enhanced tumor regression.^11^

These data have important clinical implications. Oncogenic BRAF leads to an immunosuppressive environment and treatment with a BRAFi results in an immune response that is early but transient which is likely due to the expression of immunomodulatory molecules. The addition of immune checkpoint blockade to BRAFi therapy may potentially improve responses to therapy (**Fig. 1**), although several important outstanding questions remain.

**Figure 1.**
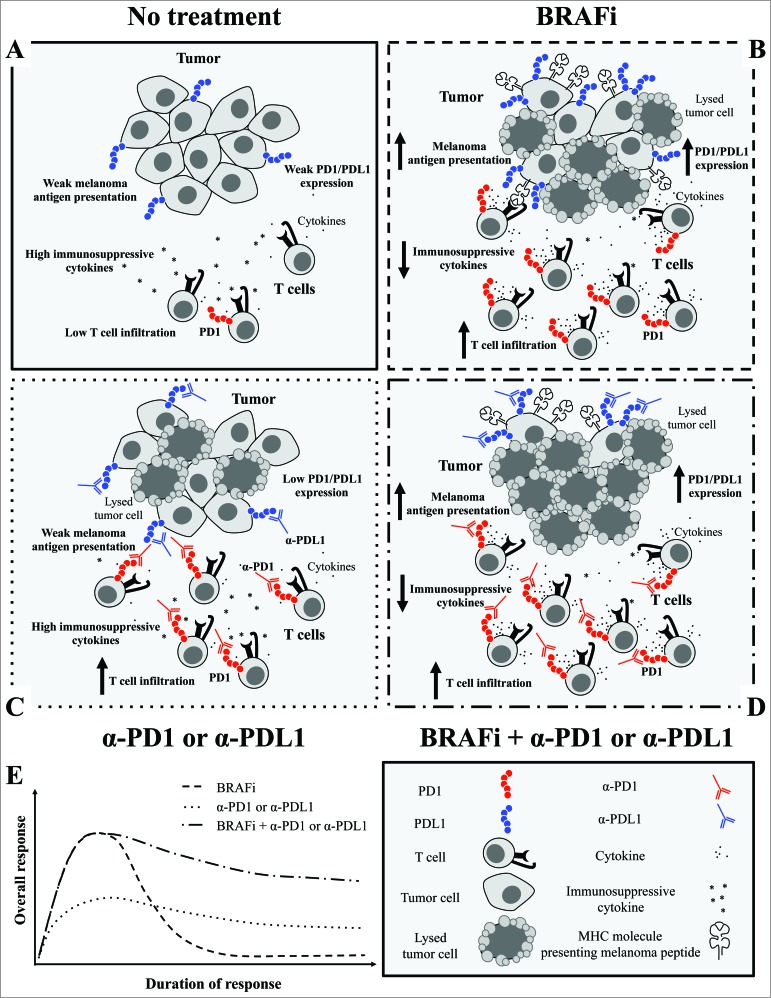
Addition of either anti-PD1 or anti-PD-L1 blocking antibody to BRAF inhibitors leads to enhanced antitumor response in melanoma. (**A**) Oncogenic BRAF contributes to immune escape through downregulation of melanoma antigens and an immunosuppressive microenvironment. (**B**) Treatment with a BRAF inhibitor results in enhanced melanoma antigen expression, a transient increase in CD8^+^ T-cell infiltrate, decreased immunosuppressive cytokines, and upregulated expression of programmed cell death 1 (PD-1) and its ligand PD-L1. (**C**) Treatment with either anti-PD-1 or PD-L1 increases T-cell infiltrate into an initially immunosuppressive environment. (**D**) Addition of anti-PD-1 or PD-L1 blocking antibody to BRAFi leads to enhanced melanoma antigen expression, a sustained increase in CD8^+^ T-cell infiltrate, decreased immunosuppressive cytokines, and a more favorable tumor microenvironment (PD-1 and PD-L1 expression are increased but are inhibited by blocking antibody) conducive to increased cancer cell death. (**E**) Together, these data suggest that BRAF-targeted therapy may synergize with immune checkpoint blockade to maximize immunologic and clinical response, and further, that the optimal timing for such immune checkpoint therapy may be early in the course of the kinase inhibitor treatment.

The sequence and timing of combination therapy is an important consideration, as there is some evidence that the immune response to BRAFi is early and transient. It is possible that there is a narrow window in which to add immune checkpoint blockade, as optimal combination therapy requires treating with immunotherapy while T cells are primed early on in the course of BRAFi. However, the addition of an immune checkpoint inhibitor at an early time-point after BRAFi initiation was associated with increased toxicity in one of the first trials combining these strategies.^9^ It is not clear if this toxicity is specific to this particular combination (vemurafenib and ipilimumab), although other unexpected toxicities have been seen using other combinations.^10^ Another question is whether adding immune checkpoint inhibitors to combined BRAF/MEK blockade will be as effective, as the mitogen activated protein kinase (MAPK) pathway is critical for T-cell activation.^6^ Studies investigating this hypothesis are currently underway.

The answers to these queries are crucial to optimize therapeutic combinations of immune checkpoint blockade with targeted therapeutics, and many of these studies are currently ongoing. Insights gained from these studies will be instrumental in guiding rational combinations of kinase inhibitors and immune checkpoint blockade for melanoma, and ultimately for other cancers.
